# Operational zoonotic containment of Middle East respiratory syndrome coronavirus in Saudi Arabia: An implementation-oriented One Health genomic framework

**DOI:** 10.14202/vetworld.2026.1322-1341

**Published:** 2026-03-28

**Authors:** Shuaibu Abdullahi Hudu, Abdulgafar Olayiwola Jimoh

**Affiliations:** 1 Center for Health Research, Northern Border University, Arar 91431, Kingdom of Saudi Arabia; 2 Department of Microbiology, Faculty of Medicine, Northern Border University, Arar 91431, Kingdom of Saudi Arabia; 3 Department of Pharmacology and Therapeutics, Faculty of Basic Clinical Sciences, College of Health Sciences, Usmanu Danfodiyo University, Sokoto, 840232 Sokoto State, Nigeria

**Keywords:** Camel vaccination, genomic epidemiology, Healthcare-associated transmission, Middle East respiratory syndrome coronavirus, One Health, zoonotic spillover

## Abstract

Middle East respiratory syndrome coronavirus (MERS-CoV) remains a persistent zoonotic threat more than a decade after its first detection, with Saudi Arabia continuing to be the global epicenter of human infections and the main reservoir interface through dromedary camels. Despite ongoing surveillance, advances in molecular diagnostics, and research on vaccines and therapeutics, sporadic zoonotic spillovers and healthcare-associated outbreaks still occur, showing that current prevention strategies are still not enough. This review compiles current evidence from epidemiological studies, camel reservoir research, genomic monitoring, and public health reports published between 2012 and April 2025 to identify the key gaps preventing effective containment. Special focus is given to recent genomic discoveries, including post-2022 clade B sublineages, recombination events, and spike protein changes that might affect transmission and the effectiveness of countermeasures. Available data suggest that MERS-CoV epidemiology is driven by repeated camel-to-human transmission, followed by occasional amplification in healthcare settings rather than sustained community spread. High seroprevalence and frequent detection of viral RNA in juvenile camels, seasonal gathering in markets, and extensive animal movement networks contribute to ongoing viral circulation at the animal–human interface. Genomic studies consistently show close phylogenetic relationships between camel and human isolates, confirming recurrent zoonotic transmissions. However, fragmented surveillance systems, delayed genomic data integration, inconsistent biosecurity practices, and limited field evidence for camel vaccination pose major barriers to control. Additionally, hospital outbreaks continue to occur due to delayed diagnosis, overcrowding, and incomplete adherence to infection-prevention protocols, underscoring the need for improved clinical preparedness. Based on the integrated synthesis of epidemiological, veterinary, and genomic evidence, this review proposes an implementation-focused One Health genomic framework tailored to the Saudi context. The proposed roadmap highlights real-time connection of human and camel surveillance, expands genomic sequencing capacity, targets vaccination strategies in camels and high-risk human populations, standardizes biosecurity measures in markets and abattoirs, and strengthens infection control systems in healthcare facilities. Alignment with national governance structures and Saudi Vision 2030 offers a practical pathway for coordinated multi-sectoral action. This review concludes that MERS-CoV is unlikely to be eradicated soon, but it can be effectively managed through a genomics-enabled, operational One Health approach that combines surveillance, vaccination, clinical preparedness, and policy coordination. The model outlined here provides a scalable way to reduce zoonotic spillover risk and strengthen readiness against future coronavirus and emerging zoonotic threats.

## INTRODUCTION

Middle East respiratory syndrome coronavirus (MERS-CoV) has remained a significant zoonotic and public health concern since its initial identification in 2012 during the emergence of novel respiratory coronaviruses [1]. Saudi Arabia continues to report the majority of confirmed human cases and related fatalities globally [2]. Although substantial improvements have been made in surveillance systems and infection prevention and control measures, MERS-CoV still causes sporadic zoonotic transmission events and recurring healthcare-associated outbreaks [3, 4]. Ongoing viral circulation in dromedary camel populations, along with frequent human–animal interactions, remains a major factor in sustaining the risk of transmission [5]. Additionally, recent epidemiological and genomic investigations have shown continuous viral evolution in Saudi Arabia, highlighting the ongoing potential for outbreak recurrence and further spread [6, 7].

This review highlights the ongoing gaps in integrating epidemiological surveillance, genomic analysis, and the operational use of the One Health approach in controlling MERS-CoV. While genomic sequencing has increasingly been utilized to clarify transmission routes, viral diversity, and outbreak patterns [8, 9], its regular integration into coordinated human, animal, and environmental surveillance remains limited. Fragmented data sharing systems, delayed genomic reporting, and insufficient translation of molecular insights into public health policies have hindered the effectiveness of current prevention efforts [10, 11]. Strengthening a genomics-based One Health framework is therefore crucial for early detection of zoonotic spillover, more targeted interventions, and better preparedness for future outbreaks in Saudi Arabia and other endemic regions [12, 13].

Despite over a decade of surveillance and extensive research on MERS-CoV, key gaps still exist in turning existing knowledge into effective, sustainable control strategies. Most studies have focused on individual aspects, such as human epidemiology, camel reservoir dynamics, viral genomic evolution, and infection prevention, but these areas are often studied separately rather than within a coordinated framework. As a result, the practical use of the One Health approach remains limited, especially in connecting epidemiological surveillance with genomic analysis across human, animal, and environmental systems. In Saudi Arabia, where most global cases are reported, surveillance for human infection, camel monitoring, and genomic sequencing are not fully integrated, which may delay detecting zoonotic spillover events and weaken targeted interventions.

Although genomic sequencing has enhanced understanding of transmission pathways, viral diversity, and outbreak dynamics, its routine use in public health decision-making remains inconsistent. Limited sequencing coverage, delayed data sharing, and incomplete integration of epidemiological and genomic information hinder the ability to identify transmission sources or link human cases to specific animal exposures. Additionally, the effectiveness of potential control measures, such as camel vaccination, targeted vaccination of high-risk human groups, and improved biosecurity practices at the animal–human interface, has not been sufficiently assessed under field conditions. Recurrent healthcare-associated outbreaks continue to occur despite advances in infection prevention and control, revealing ongoing operational weaknesses in early detection, case management, and outbreak response. These limitations emphasize the need for a comprehensive approach that synthesizes epidemiological evidence, genomic data, reservoir ecology, and public health practice within an implementation-oriented One Health framework tailored to the Saudi Arabia context.

Therefore, this review aims to provide a comprehensive and implementation-focused overview of current knowledge on MERS-CoV epidemiology, camel reservoir ecology, viral genomic evolution, and public health control measures, with particular emphasis on their integration within a One Health framework relevant to Saudi Arabia. This review seeks to evaluate available evidence from human surveillance, animal studies, genomic investigations, and outbreak reports to identify the factors responsible for ongoing zoonotic spillover and recurring healthcare-associated transmission. Special attention is given to recent genomic findings, including newly reported clade B sublineages and spike protein variations, and their potential implications for surveillance, vaccine development, and outbreak preparedness.

Additionally, this review aims to identify the operational limitations that hinder the effectiveness of current prevention strategies, such as fragmented surveillance systems, limited integration of genomic data, scarce field evidence for camel vaccination, and inconsistent implementation of infection prevention and control in healthcare settings. Based on these findings, the review proposes a Saudi Arabia-specific, genomics-informed One Health framework that highlights real-time connection between human and camel surveillance, expanded genomic monitoring, targeted vaccination strategies, enhanced biosecurity at the animal–human interface, and better preparedness in healthcare facilities. By shifting from descriptive analysis to practical recommendations aligned with national public health priorities, this review seeks to offer a realistic plan for reducing the risk of MERS-CoV spillover and strengthening readiness for future zoonotic coronavirus outbreaks.

## REVIEW METHODOLOGY

### Study design and review framework

This review was conducted using a narrative approach with structured evidence synthesis to combine diverse information from human epidemiology, camel reservoir studies, and genomic surveillance, where formal meta-analysis was not always possible due to variations in study design, outcomes, and reporting. This framework was chosen to facilitate policy-relevant interpretation of the available evidence and to support the development of an operational One Health strategy applicable to Saudi Arabia, in line with the objectives of this review.

### Data sources and literature search strategy

A systematic literature search was conducted using PubMed, Scopus, and Web of Science, complemented by targeted retrieval of World Health Organization (WHO) Disease Outbreak News, Saudi Ministry of Health reports, and technical documents from international public health agencies. The search included studies published up to April 30, 2025, employing combinations of keywords and Boolean operators such as: (“MERS” OR “MERS-CoV”) AND (“Saudi Arabia” OR “camel” OR “dromedary” OR “genomic” OR “sequence” OR “phylogeny” OR “outbreak” OR “hospital” OR “infection control” OR “vaccine” OR “therapeutics” OR “One Health”). Reference lists of selected articles were manually screened to identify additional relevant publications. Only English-language sources were included to ensure consistency in data extraction and interpretation.

### Study selection criteria

Eligible sources included peer-reviewed original studies, surveillance reports, and authoritative reviews that addressed one or more of the following areas: (i) human epidemiology, transmission, or outbreak investigation; (ii) camel reservoir evidence, including seroprevalence, viral RNA detection, and exposure pathways; (iii) genomic characterization, such as sequence-based epidemiology, lineage dynamics, and spike evolution; and (iv) therapeutics and vaccine development from preclinical to clinical stages. Exclusion criteria consisted of studies lacking sufficient methodological detail, reports unrelated to MERS-CoV epidemiology or control, duplicate datasets without additional analysis, and publications focused on other coronaviruses that are not directly relevant to MERS-CoV control in Saudi Arabia.

### Screening and selection

Titles and abstracts were screened, followed by full-text evaluation of potentially eligible articles. Screening decisions were made independently by the reviewer, and disagreements were resolved through discussion. The final evidence set was categorized into the following domains: epidemiology, camel reservoir, genomics, therapeutics and vaccines, and One Health implementation, to enable structured narrative synthesis.

### Data extraction and management

Key variables extracted from the selected studies included study location, study period, population characteristics, sample size, sampling context, outcome measures, incidence patterns, risk factors, seroprevalence or RNA detection, secondary attack rates, and genomic characteristics such as sequence availability, lineage or clade assignment, and reported mutations. These data were summarized narratively to ensure consistency across domains and to aid interpretation related to policy and implementation.

### Genomic data sources and inclusion criteria

Genomic information was gathered from studies reporting publicly available MERS-CoV sequences deposited in GenBank and GISAID databases. Included studies analyzed complete or near-complete genomes and offered clear sampling context. Special attention was given to post-2022 genomic data, newly identified clade B sublineages, recombination events, and spike mutations with potential impacts on viral fitness, transmission, and vaccine development.

### Genomic analysis and interpretation

Primary phylogenetic analyses were not conducted in this review. Instead, published genomic findings were critically analyzed and interpreted alongside epidemiological and reservoir data to enhance understanding of zoonotic spillover and to guide One Health–based control strategies.

### Epidemiological data sources

Human epidemiological data were gathered from WHO situation reports, Saudi Ministry of Health surveillance summaries, and peer-reviewed outbreak investigations. Variations in case definitions, reporting periods, and potential under-ascertainment, especially during the COVID-19 pandemic, were taken into account during interpretation.

### Camel reservoir and animal health data

Studies on camel seroprevalence, viral RNA detection, age distribution, seasonal patterns, and sampling at farms, markets, or abattoirs were reviewed to assess factors influencing zoonotic spillover risk. Evidence consistently identified dromedary camels as the main reservoir; however, the likelihood of individual camel-to-human transmission could not be measured because most data were observational and exposure was cumulative rather than discrete.

### Vaccine and therapeutics evidence assessment

Evidence related to vaccines and therapeutics was reviewed narratively based on development stages, including immunogenicity, scalability, cold-chain requirements, regulatory status, and relevance to the Saudi Arabia context, including considerations for camel vaccination approval pathways.

### Development of One Health framework

A Saudi-specific One Health genomic framework was created by integrating epidemiological, genomic, veterinary, and public health evidence, along with expert interpretation and alignment with national policy structures and Saudi Vision 2030 to ensure practical implementation.

## HISTORICAL TIMELINE AND BURDEN IN SAUDI ARABIA

Since the first human cases were identified in 2012, Saudi Arabia has remained the country most affected by MERS [6]. Global reports from the WHO and the Centers for Disease Control and Prevention up to 2023–2025 indicate approximately 2,600 confirmed cases worldwide, with most originating from Saudi Arabia [6, 14]. Although annual case numbers and case-fatality estimates have varied over time, the reported fatality proportion has consistently remained high compared with many other respiratory pathogens, ranging from about 30% to 40% [15, 16]. When adjusted for population size using World Health Organization Eastern Mediterranean Regional Office (WHO EMRO) surveillance data, Saudi Arabia recorded 1901 laboratory-confirmed MERS cases between June 2012 and December 2018, corresponding to an average annual incidence of roughly 9 symptomatic infections per million people, with notable clustering during outbreak years [17]. Fatality rates tend to be higher among older adults and males, suggesting the influence of age-related comorbidities and sex-related risk factors [18, 19]. Occupational exposure remains a key factor in infection risk, as healthcare workers, camel handlers, abattoir workers, and market-related personnel are disproportionately affected, reflecting the combined role of zoonotic exposure and healthcare-associated amplification in shaping overall disease burden [20]. Temporal patterns show sporadic cases with occasional healthcare-associated outbreaks rather than sustained community transmission. Following the major epidemics reported during 2014–2015, the number of cases declined in the late 2010s. During the early period of the COVID-19 pandemic, reported MERS cases further decreased, possibly due to changes in healthcare-seeking behavior, diagnostic priorities, or surveillance intensity [21, 22].

**Figure 1:**
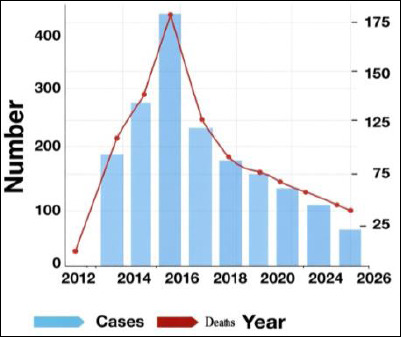
An epidemic curve of confirmed Middle East respiratory syndrome coronavirus (MERS-CoV) cases and deaths in Saudi Arabia (2012–2025), based on trend data modeled from WHO and Saudi MOH reports [2, 26]. The figure depicts annual trends in laboratory-confirmed MERS-CoV cases and related deaths, compiled from the World Health Organization Disease Outbreak News and Saudi Ministry of Health reports. The decline observed during 2020–2022 likely reflects under-ascertainment due to COVID-19-focused surveillance rather than a genuine interruption of transmission.

Nevertheless, WHO and regional surveillance reports through 2024–2025 continue to document sporadic human infections and small clusters across several regions of Saudi Arabia, with periodic reports of new cases and occasional fatalities, indicating ongoing local circulation of MERS, as shown in Figure 1 [2, 23–26]. The total number of MERS-CoV cases may seem relatively low in raw numbers but becomes more significant when adjusted for population size, with estimated rates ranging approximately from 1 to 6 cases per million people since 2012 [23]. These population-adjusted indicators highlight the continued public health importance of MERS-CoV despite the decline in yearly case counts, especially considering the high case-fatality rate. Epidemiological analysis shows relatively consistent risk across most population groups, except for those with occupational exposure. Data from the Camel–Animal–Human Network for MERS Surveillance and Control, consistent with other studies [23], suggests increased risk among individuals with frequent camel contact, including workers in farms, markets, and healthcare environments [24, 25].

In contrast, healthcare workers represent a significant portion of secondary cases, mainly due to nosocomial transmission linked to delayed diagnosis, overcrowding, or lapses in infection prevention and control during outbreaks [27]. This possible underreporting and reporting bias should be considered when analyzing temporal trends, especially during the COVID-19 pandemic. From 2020 to 2022, healthcare utilization, diagnostic capacity, and surveillance systems primarily focused on SARS-CoV-2, which may have led to underdetection of mild, asymptomatic, or community-acquired MERS-CoV infections [28]. Decreased healthcare use, diagnostic overshadowing by COVID-19, and temporary disruptions to routine surveillance likely contributed to the observed decline in reported MERS-CoV cases. These issues underscore the need to maintain comprehensive and integrated surveillance systems capable of detecting endemic zoonotic pathogens even amid concurrent public health crises [6, 29]. Overall, these epidemiological features highlight the interaction between occupational exposure, healthcare-associated amplification, and surveillance sensitivity, providing important context for understanding zoonotic spillover and transmission dynamics discussed in later sections.

**Figure 2:**
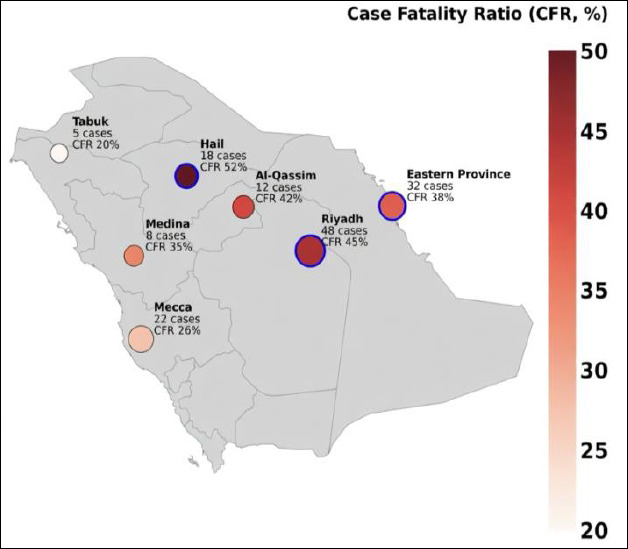
Geographical distribution of laboratory-confirmed cases of Middle East respiratory syndrome coronavirus infection in Saudi Arabia from 2022 to 2025. The bubble size indicates the total human cases by province, and color intensity shows the case-fatality ratio. Hotspot regions (Riyadh, Hail, Eastern Province) are highlighted, reflecting the overlap of camel exposure, referral hospitals, and risk of nosocomial transmission [3, 26].

The geographical distribution of MERS cases reveals a pattern linked to camel exposure regions and rural areas connected to referral pathways to tertiary hospitals (Figure 2). Hospital clusters in major urban centers, including Riyadh and Jeddah, are often associated with overcrowded emergency departments, delayed isolation, and the movement of infected patients before laboratory confirmation [30, 31]. Older individuals and those with chronic conditions such as diabetes, chronic kidney disease, and cardiovascular disease face a higher risk of severe outcomes. Healthcare workers are also disproportionately represented among reported cases and nosocomial clusters [32, 33]. Over the past decade, surveillance and outbreak investigations have enhanced case detection and contact tracing; however, the lack of standardized national outbreak response procedures and the absence of real-time integration between animal surveillance and human case reporting remain significant limitations. Reliable national case counts are crucial for effective surveillance planning, and community-level genomic monitoring is vital for understanding transmission patterns. The episodic nature of MERS cases necessitates ongoing preparedness even during periods of low incidence. Hospital-based infection prevention and control, along with rapid diagnostic capacity, remain essential for preventing amplification after spillover events. Historical experience also demonstrates that targeted measures, such as improving emergency department flow, early isolation, and staff training, can successfully break nosocomial transmission and should be maintained as standard practice.

### Camel reservoirs and zoonotic transmission

Dromedary camels are recognized as the main reservoir hosts of MERS-CoV [34]. Seroepidemiologic and molecular studies conducted across the Arabian Peninsula and Africa have consistently shown high seroprevalence in adult camels along with frequent detection of viral RNA in younger animals. Overall seroprevalence rates often exceed 70%–80%, and high polymerase chain reaction (PCR) detection rates in juvenile camels, which likely contribute to active transmission within herds, have been reported in several regions of Saudi Arabia, as summarized in Table 1 [34–36]. These findings explain why zoonotic spillover continues to happen despite the relatively low number of reported human cases, as the ecological interface between animal shedding and human exposure is repeatedly maintained, creating ongoing opportunities for transmission.

**Table 1:** Compilation of camel Middle East respiratory syndrome coronavirus seroprevalence and RNA detection in Saudi Arabia from 2012 to 2024. Surveillance studies consistently demonstrated high (>80%) seroprevalence in dromedaries, whereas RNA detection was more frequent in juveniles and during winter.

Region	Year	Sample size (n)	Seroprevalence (%)	RNA prevalence (%)	Age group	Reference
Riyadh	2012	150	85	12	Juvenile	[34]
Jeddah	2013	200	90	8	Adult	[34]
Al-Hasa	2014	120	88	15	Juvenile	[35]
Qassim	2015	180	83	10	Mixed	[35]
Riyadh	2016	210	92	14	Juvenile	[35]
Jeddah	2017	175	87	9	Adult	[35]
Hofuf	2018	140	89	16	Juvenile	[36]
Qassim	2019	190	91	11	Juvenile	[36]
Riyadh	2021	220	86	13	Mixed	[36]

Sampling was predominantly cross-sectional and convenience-based at markets, farms, or abattoirs, and the original studies reported age stratification and sample sizes.

Transmission most commonly occurs through close physical contact with camels or exposure to their respiratory secretions. Occupational risk groups include camel herders, agricultural workers, abattoir staff, and market handlers, while cultural practices such as drinking raw camel milk and maintaining close contact with camels may further increase the likelihood of human exposure [37, 38]. Temporary hotspots of exposure can arise through animal trade, movement of camels, and market aggregation, where animals from multiple locations are brought together in markets or slaughterhouses [39, 40]. Although precise estimates of per-contact spillover probability remain uncertain, epidemiological and modeling studies consistently show that zoonotic transmission risk increases with the frequency, duration, and intensity of exposure to infected camels or their secretions. Case-control and phylogenetic investigations indicate that most primary human MERS-CoV infections result from repeated or prolonged exposure rather than single-contact events, particularly among herders, shepherds, and slaughterhouse workers [41]. Modeling studies further suggest that even modest reductions in viral shedding at the animal–human interface could substantially reduce spillover events, although this effect may be offset by the high prevalence of infection in camel populations and repeated exposure of humans [42].

Camel movement networks are crucial in maintaining MERS-CoV circulation and influencing the spatial distribution of spillover risk. Seasonal gathering of camels at markets, breeding farms, and slaughterhouses boosts viral amplification within herds, especially when animals from diverse regions are mixed. Trade-related movement for racing, breeding, or slaughter can create temporary transmission hotspots and introduce infected animals into herds that were previously unexposed. These factors may partly explain why human cases tend to cluster around major camel markets and referral hospitals, despite the lack of sustained community transmission [43, 44]. Transmission at the camel–human interface is also affected by seasonal patterns. Multiple studies have shown increased viral RNA detection during cooler months, which overlap with calving seasons when juvenile camels are more common in herds [45, 46]. The age composition of camel populations is therefore a key factor in transmission intensity, as young animals serve as amplification sources of infection within herds and raise the risk of human exposure during routine husbandry or market activities. These seasonal and demographic factors offer a biological explanation for the cyclic and period-specific rises in spillover risk observed in various regions of Saudi Arabia [41, 42].

Genomic studies often show close phylogenetic links between viruses from camels and those found in later human cases, supporting camel-to-human transmission as the main source of new infections. Younger camels tend to carry higher viral RNA loads and thus play a bigger role in spreading the virus within herds. Control measures that ignore camel population structure and handling practices are less likely to succeed. Both modeling and intervention research indicate that targeted actions, like vaccinating young camels or those involved in markets, could decrease viral spread and reduce human infection risk [42]. However, despite modeling suggesting benefits of camel vaccination, there is still limited large-scale evidence on how effective, feasible, approved, and accepted such measures are among camel owners.

From a One Health perspective, integrated surveillance that combines paired animal and human sampling, genomic sequencing, and shared data platforms is crucial for detecting spillover events in near real time [47, 48]. Saudi Arabia has conducted extensive camel surveillance and genomic studies, but full integration between veterinary and human health sequencing systems has not yet been achieved. Better integration would enable more precise attribution of human cases to animal sources and could inform targeted vaccination, movement control, and cost–benefit analyses of intervention strategies. Practical considerations include culturally appropriate engagement with camel-owning communities, clear mechanisms for compensation and insurance for vaccinated animals, and logistical planning for vaccine storage and distribution in desert and rural environments. Field studies show that the availability of fencing, isolation areas, and hygiene facilities varies significantly across sites. Although veterinary surveillance has increased the detection of infected animals, biosecurity measures are not consistently implemented, especially in informal markets and small-scale operations [49, 50]. Consequently, close human–animal contact, limited use of protective equipment, and poor environmental hygiene may continue to facilitate exposure. These findings emphasize the importance of standardized biosecurity, effective risk communication, and active stakeholder involvement as fundamental parts of any One Health approach aimed at reducing zoonotic spillover. Overall, ecological, demographic, and trade-related factors influencing camel infection create the context in which viral evolution continues to drive repeated zoonotic transmissions to humans.

### Genomic characterization of MERS-CoV

Since the emergence of MERS-CoV in Saudi Arabia, genomic analysis has been crucial in understanding its epidemiology and how it evolves. Early sequencing studies showed that most human infections came from multiple independent zoonotic spillover events from dromedary camels, rather than ongoing human-to-human transmission [51]. Phylogenetic analyses identified two main lineages, clade A and clade B, with clade B quickly becoming the dominant lineage after 2013. Several sublineages within clade B have been linked to hospital outbreaks in Riyadh, Jeddah, and the Eastern Province, providing clear evidence that infections acquired in hospitals have significantly contributed to the overall disease burden [52, 53]. Viral RNA has also been found in camels with high seroprevalence, and paired human–camel samples frequently show nearly identical genomes, supporting repeated transmission from animals to humans. Human outbreaks mostly result from spillover of different camel viruses rather than the continuous circulation of a single lineage, which explains the sporadic but recurrent outbreaks seen in various parts of Saudi Arabia.

Two key features define the evolution of MERS-CoV. First, the substitution rate is relatively slow, aligning with its enzootic maintenance in camel populations, which allows for estimating introduction times and tracing outbreak paths. Second, recombination occurs often, creating mosaic genomes that complicate phylogenetic analysis. Recombination events have been observed in several clade B sublineages and are considered a major source of genetic diversity [54]. Because of this, whole-genome sequencing is essential for accurate outbreak investigation and molecular epidemiology. The spike (S) glycoprotein, which binds to the dipeptidyl peptidase-4 (DPP4) receptor and is the primary target of neutralizing antibodies, has been extensively examined in functional genomic studies [55]. Multiple amino acid changes in the S-RBD and S2 regions have been identified in naturally circulating strains [56]. Mutations like D510G and I529T have demonstrated reduced viral entry efficiency in in vitro studies, implying that some variants may impact host cell infectivity [57–59]. Still, additional mutations found in more recent lineages, especially in the B.5 cluster identified during 2023–2024 surveillance, affect regions involved in receptor binding and membrane fusion [45, 60]. The phenotypic effects of these changes are still being studied, underscoring the importance of combining genomic surveillance with laboratory testing.

Although genomic sequencing has significantly enhanced understanding of MERS-CoV evolution, the number and representativeness of available genomes still remain limited compared to the total number of human and camel infections. Sequenced isolates account for only a small fraction of confirmed cases and sampled animals, with a bias toward outbreak-related samples rather than sporadic or rural cases. This under-sampling limits detailed reconstruction of transmission chains and may mask local viral diversity. Nevertheless, available evidence consistently shows that the epidemiology of MERS-CoV in Saudi Arabia is driven by repeated zoonotic introductions rather than sustained human transmission [16, 45]. Most genomic data from Saudi Arabia have been generated using next-generation sequencing platforms, mainly Illumina short-read technology, while fewer sequences have been obtained through alternative platforms. Most studies analyzed whole or near-complete genomes with high coverage; however, sequencing depth, completeness criteria, and quality-control standards were not always specified. Although partial genomes and amplicon-based sequences are useful for lineage assignment, they provide limited information for detecting recombination or subtle evolutionary changes, highlighting the importance of whole-genome sequencing for surveillance and outbreak investigation [61, 62].

Delays between sample collection, sequencing, and depositing data in public repositories such as GenBank or GISAID also diminish the usefulness of genomic information for real-time public health response. In several cases, sequences were uploaded months or years after sampling, reducing their value for immediate outbreak analysis. These delays emphasize the need for integrated One Health systems with coordinated workflows connecting diagnostic laboratories, sequencing facilities, and data sharing platforms to support near real time genomic surveillance [63, 64]. Combining phylogenetic findings with epidemiological data and camel movement information has yielded important insights into transmission routes. Human viruses often cluster with contemporaneous camel isolates from the same region, supporting direct zoonotic transmission. However, incomplete linkage between human and camel datasets and limited information on animal movement networks hamper the ability to connect individual spillover events to specific exposure settings. Enhancing such integration is crucial for translating genomic findings into targeted intervention strategies [42, 65].

Recent surveillance conducted between late 2023 and early 2024 in Saudi Arabia detected a high proportion of PCR-positive animals, with genomes clustering within emerging clade B lineages, including B.5-like variants [7]. These viruses contained new spike substitutions that raise concerns about antigenic drift. Laboratory studies are ongoing to determine whether these changes affect receptor binding, membrane fusion, or antibody recognition [66]. These findings highlight the importance of combining field genomics with functional studies. Three key genomic observations are especially relevant for public health. First, ongoing spillover from camels remains the main driver of human infection. Second, recombination and continuous nucleotide substitutions require ongoing genomic surveillance to identify variants with increased epidemic potential. Third, diversity in the spike protein may impact the effectiveness of medical countermeasures. Vaccines based on spike antigens, including GLS-5300, ChAdOx1-MERS, and MVA-MERS-S, along with monoclonal antibodies, must therefore be evaluated against currently circulating strains [7, 67, 68]. The continuous evolution of the virus underscores the need for periodic reassessment of these countermeasures, as demonstrated by recent clade dynamics observed in Saudi Arabia.

Amino acid substitutions and recombination within the spike glycoprotein, especially in clade B sublineages, may affect viral fitness, receptor interaction, and immune recognition. Experimental studies have demonstrated that certain mutations can change entry efficiency or neutralization sensitivity, although their impact on transmissibility and disease severity in humans remains only partly understood [68, 69]. The appearance of new clade B variants highlights the need for ongoing genomic surveillance to track circulating strains and to inform updates of vaccines and antibody-based therapies [7, 70]. Overall, genomic evidence supports the idea that repeated zoonotic introductions combined with healthcare-associated amplification drive MERS-CoV epidemiology in Saudi Arabia. However, current genomic sampling density and metadata completeness are still insufficient to accurately reconstruct detailed transmission chains or to confidently link individual human infections to specific camel exposure events.

## HUMAN EPIDEMIOLOGY AND TRANSMISSION DYNAMICS

MERS-CoV infection in humans can be asymptomatic or mild, but it may also advance to severe respiratory failure and death. Severe cases are more common in older individuals, adults with underlying conditions such as diabetes mellitus, chronic kidney disease, and cardiovascular disease, pregnant women, and immuno-compromised patients, all of whom face a higher risk of poor outcomes [71]. Although community transmission is usually limited, nosocomial transmission has repeatedly led to large outbreaks (Table 2) [72–76]. These outbreaks are often linked to predictable factors, including delayed recognition of index cases, overcrowded emergency departments, insufficient triage and isolation facilities, and patient movement between wards before diagnostic confirmation, especially during times of concurrent respiratory disease outbreaks like COVID-19.

**Table 2:** Large healthcare-associated Middle East respiratory syndrome coronavirus outbreaks in Saudi Arabia from 2013 to 2020, showing primary nosocomial outbreaks, magnitude, and gaps in infection prevention.

Year	Location	Total cases	Infected healthcare workers	Suspected IPC breach	Duration (weeks)	Reference
2013	Al-Hasa (Eastern Province hospitals)	23	2	Transmission in dialysis units, intensive care unit, wards; delayed recognition	8	[72]
2014	Jeddah (multisite hospital-linked outbreak)	255	40	Overcrowding, multiple hospital exposures, delayed recognition of cases	20	[73]
2014	Riyadh (King Fahad Medical City and other hospitals)	45	23	Delayed triage and intra-hospital spread	8	[74]
2015	Riyadh (King Abdulaziz Medical City)	130	43	ER crowding, delayed isolation, patient movement across wards	10	[75]
2017	Emergency room outbreak (study cohort)	52	45	Initial IPC lapses; improved control after strict IPC	10	[76]

IPC = infection prevention and control, HCW = healthcare worker, ER = emergency room, ICU = intensive care unit. Case counts are derived from outbreak investigation reports using hospital-based case ascertainment and contact tracing, and therefore reflect detected cases rather than population-based surveillance.

Transmission dynamics are affected by the level of viral shedding from camels or infected humans, patterns of household and healthcare contacts, and environmental factors like enclosed spaces and crowding [77, 78]. Household secondary attack rates are usually low, which explains the lack of sustained community transmission. In contrast, hospital settings may foster conditions for amplification, where aerosol-generating procedures, the presence of vulnerable patients, and insufficient use of personal protective equipment can result in high secondary attack rates among both patients and healthcare workers [33, 79]. Early case detection is therefore crucial to enable quick implementation of standard, droplet, and contact precautions, along with airborne precautions during aerosol-generating procedures, to break the chain of transmission.

Surveillance data show that many community cases are linked to recent contact with camels or visits to camel markets, indicating that most primary human infections are true zoonotic events rather than ongoing human transmission chains [40, 80]. Healthcare workers often experience clusters of cases but frequently acquire the infection from unrecognized index cases. Implementing standardized triage algorithms and screening for severe acute respiratory illness during times of increased camel infections may help reduce delays in diagnosis. Additionally, having rapid point-of-care tests available and raising clinical awareness, especially in peripheral or rural hospitals where camel-exposed patients may first seek care, could help prevent nosocomial spread [81, 82].

Quantitative analyses show that secondary transmission of MERS-CoV depends heavily on the context. Household secondary attack rates are generally low, around 3%–10%, while nosocomial attack rates can reach 20%–40% among exposed patients and HCWs when infection control measures are insufficient [83, 84]. These differences indicate that large outbreaks are mainly driven by hospital amplification rather than community spread, even when zoonotic introductions are rare. Several major outbreaks in Saudi Arabia have been linked to superspreading events, where a small number of infectious individuals caused a disproportionate number of secondary cases. These events were often linked to delayed recognition, overcrowded emergency departments, extended patient movement between wards, and lack of proper airborne precautions during aerosol-generating procedures. These findings align with the transmission traits of coronaviruses and help explain the episodic but severe outbreaks seen in healthcare settings [16, 23].

Although transmission chains in the community are usually short, healthcare settings can sustain transmission for extended periods because of high-risk procedures, frequent close contact, and the presence of vulnerable patients. As a result, the overall burden of MERS-CoV remains relatively low at the population level but can become significant when amplification occurs in hospitals after zoonotic spillover [7, 26, 85]. The course of outbreaks is strongly affected by the effectiveness of screening and triage. Delayed recognition of MERS-CoV infection in patients with acute respiratory illness, especially in non-tertiary hospitals, has repeatedly been linked to nosocomial transmission [1]. Implementing standardized triage protocols, rapid molecular diagnostics, and early isolation has been shown to reduce secondary transmission, although differences in diagnostic capacity and preparedness continue to pose challenges [26].

Mass gatherings like the Hajj and Umrah pose additional potential transmission risks in Saudi Arabia [86]. Although sustained transmission has not been observed during these events, the gathering of millions from different regions demands ongoing vigilance. Implementing enhanced surveillance, quick case detection, and strict infection control measures likely helped prevent major outbreaks, underscoring the importance of preparedness and coordinated response in lowering national and global risks [87, 88]. These transmission patterns directly impact the prioritization of preventive and therapeutic strategies, especially in healthcare settings and high-risk groups.

## THERAPEUTICS AND VACCINE DEVELOPMENT

Treatment for established MERS remains mainly supportive. In severe cases needing intensive care, management typically includes oxygen therapy, organ support, and treatment for secondary infections and complications [89]. Several antiviral and immunomodulatory agents, such as interferons, lopinavir, and monoclonal antibodies, have been tested in small clinical trials and compassionate-use programs, but results have been mixed, and none are considered standard therapy based on strong randomized controlled trial evidence [90]. In preclinical and early clinical studies, monoclonal antibodies targeting the spike protein have shown potential as additional therapy for post-exposure prophylaxis or early treatment, though their large-scale use is limited by production costs and logistical challenges. Vaccine development has advanced slowly, with several platforms, including DNA vaccines (GLS-5300) and adenoviral-vectored vaccines (ChAdOx1-MERS and MVA-MERS-S), showing immune responses in humans and completing Phase I trials with acceptable safety, [91]. Larger Phase II studies, along with collaborative efforts led by groups like the Coalition for Epidemic Preparedness Innovations (CEPI), aim to speed up vaccine development and create platforms that can be quickly used during outbreaks [92]. Trials like the Oxford/Liverpool ChAdOx1 study, started in 2023, have renewed interest in human vaccine research, but no vaccine has yet finished late-stage efficacy testing or received approval.

Currently, no antiviral treatment has consistently proven effective against MERS-CoV, and management remains mainly supportive. Interferon, lopinavir/ritonavir, and other repurposed drugs have produced mixed results in observational studies and small trials, but many lacked enough statistical power for routine clinical use. Spike-specific monoclonal antibodies have shown strong neutralizing activity in preclinical models and early clinical studies, indicating potential for post-exposure prophylaxis or early intervention in high-risk individuals; however, their widespread use is limited by cost, production capacity, and distribution challenges. Therefore, evidence for therapeutic options remains limited, and well-designed randomized controlled trials should be integrated into outbreak preparedness plans [90]. Various vaccine platforms have been explored, including DNA, viral vector, subunit, and mRNA technologies. DNA and viral-vectored vaccines have demonstrated good safety profiles, sufficient immunogenicity, and relatively simple cold-chain requirements, making them the most advanced options for use in endemic regions. In contrast, mRNA vaccines enable quick adaptation to emerging variants but currently need more complex storage conditions and higher production costs, which could restrict their deployment in resource-limited settings. These platform-specific factors are crucial for designing vaccination strategies in Saudi Arabia and other endemic areas [91].

Vaccination of dromedary camels is an additional approach to reduce viral transmission at the animal–human interface. Experimental vaccines given to camels have been shown to decrease nasal viral shedding under controlled conditions, indicating potential for lowering spillover risk. However, widespread use requires regulatory approval, evidence from field studies of effectiveness, and acceptance by camel owners and related industries. In Saudi Arabia, approving camel vaccination programs would need coordination between veterinary authorities, agriculture ministries, and public health agencies, along with assessments of safety, immunity duration, and cost-effectiveness in real-world settings. The lack of large-scale field trials and formal licensing pathways remains a significant barrier to implementation [93]. Because outbreaks are infrequent and unpredictable, typical efficacy trials are challenging to conduct, making ethical considerations crucial when evaluating vaccines and treatments. Adaptive trial designs, emergency use authorization, and targeted vaccination of high-risk occupational groups have been proposed as more practical options. These methods require careful ethical oversight to weigh potential benefits against safety and efficacy uncertainties, while ensuring fair access and clear communication with affected communities [92].

Vaccination of camels has also been proposed as a practical upstream intervention. Experimental studies show that vaccination can reduce viral shedding in camels and thereby decrease the risk of human infection. Modeling studies suggest that targeted vaccination strategies, such as immunization at market entry or vaccinating juvenile camels, could significantly lower the number of human cases; however, few field studies have evaluated effectiveness, cost–benefit, or acceptability under real-world conditions (Table 3) [91, 93–96]. Important operational issues remain unresolved, including cold-chain requirements, acceptance by camel owners, compensation for vaccinated animals, and the duration of protection in field settings. These questions should be addressed through well-designed field trials, ideally within a One Health framework that assesses both animal and human outcomes. Post-marketing surveillance will also be necessary to evaluate long-term effectiveness, durability of immunity, and rare adverse effects of vaccines or monoclonal antibodies. Integrating pharmacovigilance with genomic surveillance could enable early detection of vaccine-escape variants, allowing for rapid updates to countermeasures. Incorporating such monitoring into national surveillance systems would enhance preparedness and help sustain the effectiveness of interventions against emerging viral lineages. Overall, these therapeutic and vaccination challenges highlight the need for an integrated One Health strategy that combines antiviral development, immunization, surveillance, governance, and operational capacity.

**Table 3:** The Middle East respiratory syndrome coronavirus vaccine in the pipeline and its relevance to Saudi Arabia (2025): Human and camel vaccine candidates, trial stage findings, and spillover-reduction modeling results in Saudi Arabia.

Vaccine platform	Target	Developer	Trial stage	Key findings	References
Viral vector (ChAdOx1)	Human	University of Oxford/KSA MoH	Phase 2b (completed)	Strong neutralizing antibody response and acceptable safety profile in the KSA population	[91]
DNA vaccine (GLS-5300)	Human	GeneOne Life Science/Inovio	Phase 1/2a (long-term follow-up)	Demonstrated immunogenicity; stable at room temperature; advantageous for logistics	[94]
NCT04170829	Human	—	Clinical trial registration	Follow-up clinical evaluation of GLS-5300 vaccine	[94]
Viral vector (MVA-MERS-S)	Human	German Center for Infection Research (DZIF)	Phase 1 (ongoing)	Potent T-cell and antibody responses in early studies; evaluated as heterologous boost	[95]
Subunit (S1 protein)	Camel	KSA Agricultural Ministry/Utrecht University	Field efficacy trials	Significantly reduces nasal viral shedding in dromedary camels; immunity duration >12 months	[93]
mRNA vaccine	Human	Moderna/CEPI	Preclinical	Rapidly adaptable platform with strong preclinical immunogenicity data	[96]

KSA = Kingdom of Saudi Arabia, CEPI = Coalition for Epidemic Preparedness Innovations, MVA = modified vaccinia Ankara, ChAdOx1 = chimpanzee adenovirus Oxford 1, RCT = randomized controlled trial. Clinical and field data are derived from phase-specific trials or experimental studies, whereas spillover-reduction estimates are based on mathematical modeling rather than empirical population sampling.

## SAUDI ONE-HEALTH ROADMAP FOR MINIMIZING ZOONOTIC SPILLOVER RISK

**Figure 3:**
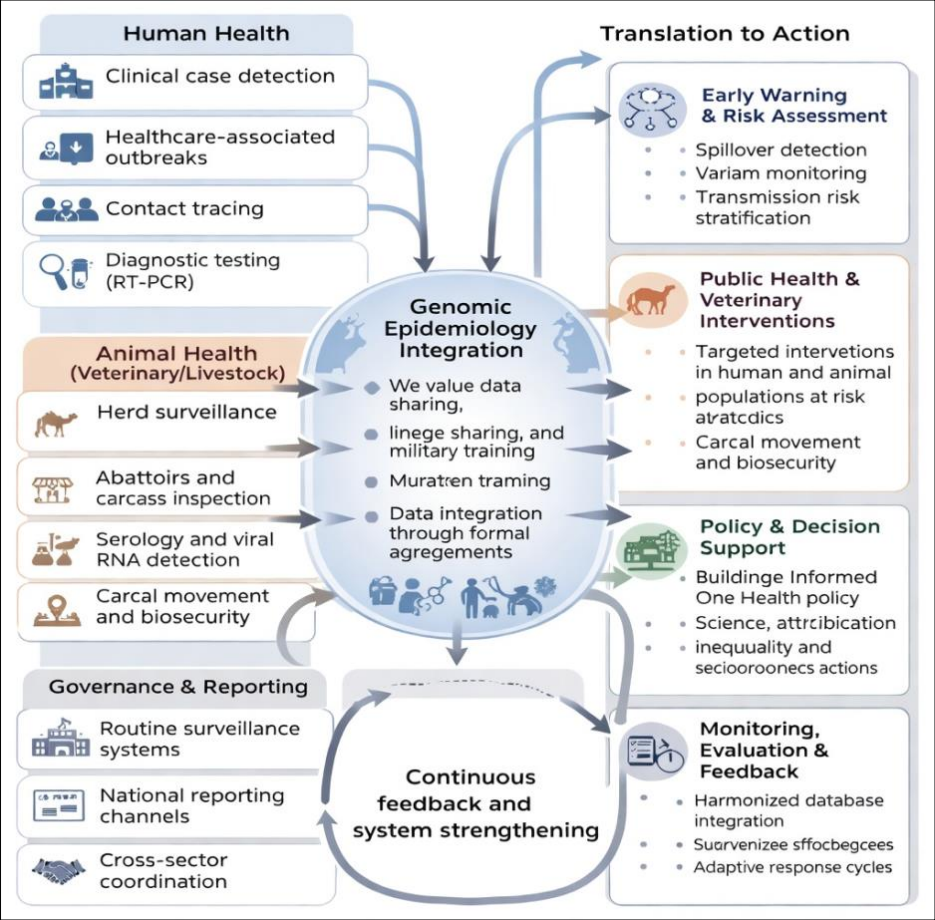
Conceptual One-Health genomic pipeline for Middle East respiratory syndrome coronavirus prevention and control in Saudi Arabia.The framework illustrates integrated surveillance of human, camel, and environmental systems linked through genomic epidemiology, enabling early spillover detection, monitoring of viral evolution, and translation of evidence into coordinated public health, veterinary, and policy responses within national One-Health governance structures [11–13, 47, 48].

A coordinated One-Health approach that integrates human health, veterinary medicine, environmental science, and public health policy is crucial for reducing zoonotic spillover of MERS-CoV in Saudi Arabia [45]. Ongoing circulation of the virus in dromedary camels, repeated spillover into humans, and amplification within healthcare settings illustrate the complex ecological interface where zoonotic pathogens emerge. Therefore, an operational roadmap should focus on continuous animal–human surveillance, enhanced biosecurity measures, and targeted risk communication programs. The integrated One Health genomic framework proposed for MERS-CoV prevention and control in Saudi Arabia is illustrated in Figure 3 [11–13, 47, 48].

Comprehensive genomic surveillance of camel populations in high-density regions such as Riyadh, Qassim, and the Eastern Province should be conducted using age-stratified sampling, given the higher viral RNA detection rates in juvenile animals. These data should be combined with routine sequencing of human cases to create matched datasets capable of identifying spillover pathways and detecting early viral evolution. Uploading sequences to national and international repositories would improve transparency and allow for global comparison of circulating strains. Enhanced biosecurity practices across camel production and trade networks are equally vital, including regulated animal movement, hygiene enforcement at markets and slaughterhouses, and vaccination against co-circulating respiratory pathogens that may increase viral shedding.

Risk communication programs aimed at camel owners, herders, traders, and abattoir workers should promote safe handling practices, discourage high-risk behaviors such as drinking raw camel milk, and encourage early reporting of suspected animal illnesses. Environmental factors must also be included in the strategy, as seasonal changes, climate conditions, and market activity influence transmission opportunities. Effective coordination among the Ministries of Health, Agriculture, Environment, and Pilgrimage Affairs is crucial, especially because the annual influx of millions of pilgrims creates unique challenges for surveillance and infection control.

Implementation of the One-Health roadmap requires measurable indicators and defined benchmarks. Key metrics should include the proportion of reported human MERS-CoV cases with available genomic sequences, the coverage of genomic surveillance in camel populations within high-risk regions, and the time interval between case detection and the availability of genomic data for public health action. Establishing surveillance targets and response timelines will enable ongoing monitoring of progress and swift adjustments during periods of increased movement or mass gatherings [48].

Clear institutional roles are essential for effective coordination. The Ministry of Health should lead human surveillance and outbreak response, supported by the National Center for Disease Prevention and Control, while veterinary authorities oversee camel surveillance, vaccination trials, and enforcement of biosecurity regulations. Environmental monitoring, sanitation, and waste management activities should involve municipal and environmental agencies. Clearly defined inter-ministerial coordination mechanisms are necessary to avoid fragmentation and ensure rapid data sharing between sectors [45].

Successful execution of the roadmap will require ongoing investment in human resources, laboratory capacity, and integrated data systems. Priorities include expanding sequencing capabilities, training multidisciplinary teams that connect human and animal health sectors, and developing interoperable databases that link epidemiological, genomic, and veterinary information. Integrating with existing national research and health programs may enhance efficiency and ensure long-term sustainability [48].

Environmental health is a vital but often overlooked aspect of MERS-CoV control. Seasonal patterns, market density, animal housing conditions, and environmental sanitation can affect the survival of the virus at the animal–human interface. Regular environmental monitoring at camel markets and slaughterhouses, along with strict hygiene enforcement, would enhance prevention efforts and support broader One-Health principles [47].

Within the healthcare sector, lessons learned from previous hospital outbreaks must inform preparedness efforts. Investments in rapid diagnostics, systematic screening of severe acute respiratory infections, and strict adherence to infection prevention and control measures remain crucial. Genomic information should steer adaptive clinical strategies, including the assessment of monoclonal antibodies and vaccine options. Long-term preparedness should also promote dual vaccination approaches, combining camel-targeted vaccines to lower reservoir viral load with human vaccination of high-risk occupational groups and healthcare workers. Modeling studies suggest that camel vaccination could significantly reduce spillover risk, although implementation will require pilot trials, regulatory approval, and stakeholder support.

Saudi Arabia is well positioned to lead such translational efforts through the National Center for Disease Prevention and Control and university-based research networks working together with CEPI and WHO. Strengthening laboratory infrastructure, training interdisciplinary teams, and developing digital dashboards linking real-time data on camel health, human infections, and environmental conditions will improve decision-making capacity. Incorporating One-Health priorities into Saudi Vision 2030 provides political support and a sustainable funding framework.

An incremental implementation strategy with clear short-, medium-, and long-term objectives will enable ongoing evaluation and adjustments. Immediate priorities include strengthening genomic links between human and camel surveillance, harmonizing biosecurity practices, conducting pilot camel vaccination trials, and expanding environmental monitoring programs [97]. Regular assessment against predefined indicators will allow adaptive modifications to the roadmap in response to changing epidemiological and genomic evidence [42, 45, 47]. Successful implementation will ultimately depend on sustained political commitment, multisectoral collaboration, and continuous monitoring, as discussed further in the Expert Opinion and Future Perspectives section.

## ETHICAL AND SOCIAL CONSIDERATIONS

Successful implementation of a One Health–based surveillance and control strategy for MERS-CoV must consider ethical, cultural, and social factors that can influence acceptance, compliance, and long-term sustainability. In regions where human–camel interactions are closely tied to livelihood, tradition, and cultural identity, risk communication strategies need to be culturally sensitive, transparent, and non-stigmatizing toward groups often involved in camel contact, including camel owners, herders, abattoir workers, and healthcare personnel [76, 77]. Messaging that implicitly blames camels or specific occupational groups may reduce trust, discourage reporting, and ultimately weaken the effectiveness of surveillance [77, 81].

Ethical governance of data sharing across human, animal, and environmental health sectors is crucial for the responsible use of genomic surveillance. Pathogen genomic data should be managed within harmonized regulatory frameworks that allow rapid sharing for public health action while safeguarding institutional, national, and personal data sovereignty [98, 99]. Transparent oversight mechanisms are essential to promote cross-sectoral and cross-border collaboration, especially when real-time genomic epidemiology becomes a routine part of public health surveillance systems [98].

Acceptance of vaccination programs, including potential camel vaccination campaigns and targeted immunization of high-risk human populations, must consider traditional practices, economic implications, animal-welfare concerns, and religious perspectives [91]. Engaging community leaders, veterinary authorities, and public health agencies is essential to address concerns about animal livelihoods, vaccine safety, and new biomedical technologies. Incorporating ethical reflection, community participation, and culturally appropriate communication into surveillance and intervention programs enhances public trust and helps maintain the effectiveness of MERS-CoV prevention and control within a One Health framework [77, 98].

## EXPERT OPINION AND FUTURE PERSPECTIVES

Based on accumulated evidence and expert assessment, MERS-CoV remains a persistent zoonotic threat despite more than a decade of surveillance, research, and outbreak response. Continued camel-to-human transmission, combined with short-term amplification in healthcare settings and the broader global vulnerability to emerging respiratory viruses, indicates that control strategies should shift from fragmented, sector-specific interventions to coordinated and operational One Health integration [33, 47]. Experience with other endemic zoonoses suggests that the highest return on investment is achieved when interventions are prioritized based on public health benefit and feasibility rather than implemented simultaneously without clear sequencing [42, 47].

Immediate priorities with high feasibility and strong impact include enhancing infection prevention and control practices in healthcare facilities, standardizing triage and rapid diagnostic protocols for severe acute respiratory infections, and routinely integrating epidemiological surveillance with viral genomic data [76, 77, 79]. These measures can be adopted within existing healthcare systems and have consistently proven effective in preventing healthcare-associated amplification, which remains the main driver of large MERS-CoV outbreaks [72, 73, 79]. Failing to maintain these core precautions could lead to recurrent outbreaks with significant clinical, economic, and reputational consequences [33, 77].

Medium- to long-term interventions that are moderately feasible but have significant impact include expanding systematic genomic surveillance in dromedary camels, integrating veterinary and human surveillance systems, and standardizing biosecurity practices in camel markets and abattoirs [38, 47]. These measures directly target upstream factors of zoonotic spillover and align with successful One Health programs where integrated surveillance combined with targeted risk reduction at the source has led to sustained decreases in animal-to-human transmission [42, 47]. Pilot camel vaccination programs also show promise in reducing viral shedding, though evidence of field effectiveness is limited, and regulatory guidance, stakeholder acceptance, and operational assessment are needed before large-scale deployment [93].

More resource-intensive strategies, such as nationwide camel vaccination or mass human immunization, should be guided by evidence obtained through phased implementation and operational research rather than immediate large-scale deployment [91, 100]. Premature expansion of such programs without sufficient data may lead to inefficient use of resources and limited public health benefits. Ongoing fragmentation of human, animal, and environmental surveillance systems would likely allow continued sporadic spillover events followed by costly healthcare-associated outbreaks, potentially undermining the effectiveness of downstream medical counter-measures [47, 48].

Future research priorities should therefore be sequential and implementation-focused. Short-term goals include increasing genomic sampling density, especially for paired human–camel cases, and assessing the real-world effectiveness of hospital-based surveillance and triage systems [47]. Medium-term priorities involve advanced field testing of camel vaccines, evaluating biosecurity measures in markets and abattoirs, and creating interoperable data platforms that connect veterinary and human health information systems [42, 93]. Long-term considerations should focus on cost-effectiveness, duration of protection, and the acceptability of dual-strategy vaccination approaches targeting both camel reservoirs and high-risk human groups [42, 91].

Saudi Arabia holds a unique position in global MERS-CoV preparedness due to its disease burden, governance capacity, regulatory infrastructure, and experience in managing infectious disease risks during large mass gatherings [38]. A Saudi-specific One Health genomic framework that integrates surveillance, genomics, healthcare preparedness, and veterinary interventions could serve as a model for controlling other zoonotic viral diseases and emerging pathogens [45, 47]. Ultimately, the ability to turn the One Health concept into sustained operational practice will determine whether MERS-CoV remains a sporadic, high-impact threat or becomes a manageable zoonosis with minimal disruption to public health.

## CONCLUSION

This review synthesizes current evidence on the epidemiology, zoonotic transmission, genomic evolution, healthcare-associated amplification, and prevention strategies of MERS-CoV in Saudi Arabia, emphasizing the ongoing public health importance of the virus despite decreasing annual case numbers. Available data consistently show that the epidemiology of MERS-CoV is driven by repeated zoonotic spillover from dromedary camels, followed by episodic amplification in healthcare settings rather than sustained community transmission. High seroprevalence in camels, frequent viral detection in juvenile animals, and genetic similarity between camel and human isolates support the animal reservoir as the main source of infection. Genomic analyses further indicate that most outbreaks stem from independent introductions, with clade B sublineages dominating recent years and occasional recombination events contributing to viral diversity. Healthcare-associated outbreaks continue to be the primary factor influencing outbreak size, often linked to delayed diagnosis, overcrowding, and lapses in infection prevention and control. Therapeutic options remain limited to supportive care, and although several vaccine platforms and monoclonal antibodies show potential, no licensed human or camel vaccine is currently available. Overall, these findings highlight that MERS-CoV remains a low incidence but high-impact zoonosis requiring continuous surveillance and coordinated control measures.

From a practical standpoint, the evidence strongly supports prioritizing interventions that are feasible to implement and can deliver immediate public health benefits. Enhancing infection prevention and control in healthcare settings, improving triage and rapid diagnostic capabilities, and ensuring routine links between epidemiological surveillance and genomic data remain the most effective strategies for preventing large outbreaks. Upstream measures, including systematic monitoring of dromedary camels, better biosecurity in markets and slaughterhouses, and targeted risk communication among high-exposure occupational groups, are essential for reducing spillover risk. Integrating human, animal, and environmental data within a One Health approach offers the most realistic pathway for early detection, prompt response, and informed policymaking. Given Saudi Arabia’s high camel density, extensive animal trade networks, and large annual gatherings, such integration is especially critical.

This review has several strengths. It synthesizes epidemiological, genomic, veterinary, and clinical evidence within a unified One Health approach, enabling a comprehensive understanding of transmission dynamics and control priorities. The inclusion of recent genomic findings, vaccine development data, and operational factors relevant to Saudi Arabia offers a policy-focused summary rather than just a descriptive overview. Additionally, the review emphasizes the importance of connecting surveillance data with implementation strategies, which is crucial for turning scientific knowledge into effective public health actions.

However, several limitations should be recognized. Much of the current evidence comes from observational studies, outbreak investigations, and modeling analyses, which may be affected by underreporting, sampling bias, and incomplete genomic data. Human and camel surveillance information are not always collected at the same time, which limits the ability to accurately trace transmission chains. Genomic sequences are available for only a small percentage of cases, and delays in sequencing and data sharing reduce the usefulness of molecular data for real-time response. Evidence on vaccines and therapeutics is still mainly based on early-phase trials or experimental studies, and field data on camel vaccination, biosecurity measures, and long-term effectiveness are limited. These gaps hamper the ability to fully assess the impact of proposed control strategies.

Future research should prioritize implementation-focused efforts over purely descriptive studies. Enhancing the frequency and timeliness of genomic sampling, especially for paired human–camel cases, will deepen understanding of spillover dynamics. Field assessments of camel vaccination, biosecurity measures, and integrated surveillance systems are necessary to evaluate their cost-effectiveness and practicality in real-world settings. Creating interoperable databases that connect human, animal, and environmental surveillance will be crucial for operational One Health initiatives. Additionally, establishing adaptive clinical trials and preparedness platforms will enable quick testing of vaccines and therapeutics during outbreaks. Long-term strategies should also address the acceptability, economic impact, and sustainability of dual approaches targeting both animal reservoirs and high-risk human populations.

In conclusion, MERS-CoV remains an endemic zoonotic threat in Saudi Arabia that cannot be eliminated through single-sector interventions alone. Evidence gathered over the past decade shows that effective control requires ongoing genomic surveillance, enhanced healthcare readiness, better management of animal–human interfaces, and coordinated One Health governance. Saudi Arabia is uniquely positioned to lead the development of an operational One Health model that integrates surveillance, genomics, veterinary actions, and public health responses. If successfully implemented, such a framework could not only lessen the impact of MERS-CoV but also serve as a model for preparedness against future zoonotic and emerging infectious diseases.

## DATA AVAILABILITY

All the generated data are included in the manuscript.

## AUTHORS’ CONTRIBUTIONS

SAH: Designed the study, analyzed the data, and drafted the manuscript. AOJ: Interpreted the data and critically revised the manuscript. Both authors have read and approved the final version of the manuscript.
